# Supplementary material for: Multiscale coupling of surface temperature with solid diffusion in large lithium-ion pouch cells
Source: Commun Eng. 2022 May 26;1:1. doi: 10.1038/s44172-022-00005-8 (PMC10938860; doi:10.1038/s44172-022-00005-8)
Supplement: Supplementary file 1 — Supplementary Information [file 44172_2022_5_MOESM1_ESM.pdf]

Supplementary Information

Multiscale coupling of surface temperature with solid diffusion in large lithium-ion pouch cells

Jie Lin[a], Howie N. Chu[a], David A. Howey[a,b], Charles W. Monroe[a,b,*]

[a]*Department of Engineering Science, University of Oxford, Oxford, OX1 3PJ, United Kingdom*

[b]*The Faraday Institution, Harwell Campus, Didcot, OX11 0RA, United Kingdom*

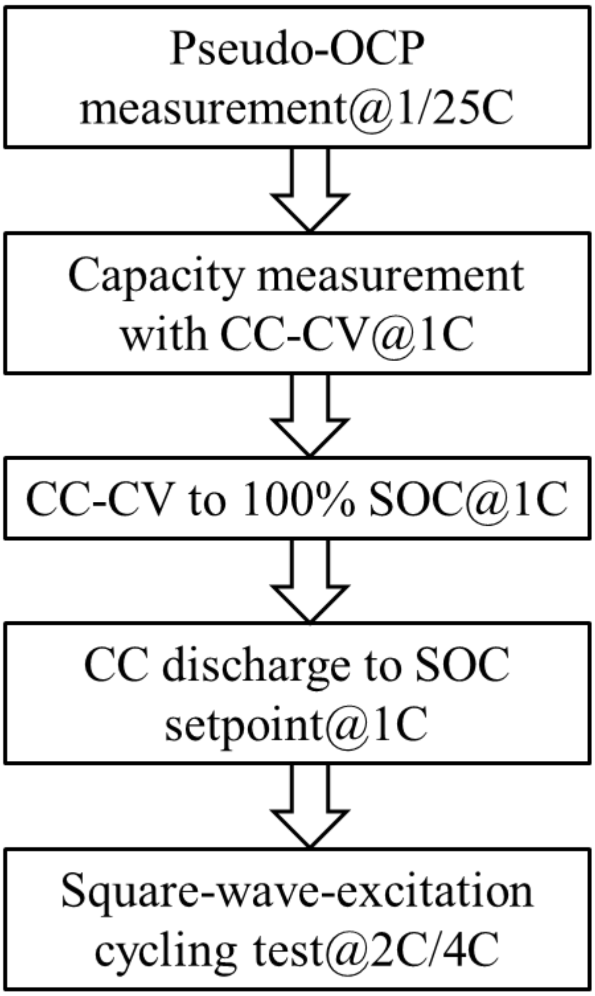

Figure S1. Test protocol for square-wave current excitation of LFP pouch cells.

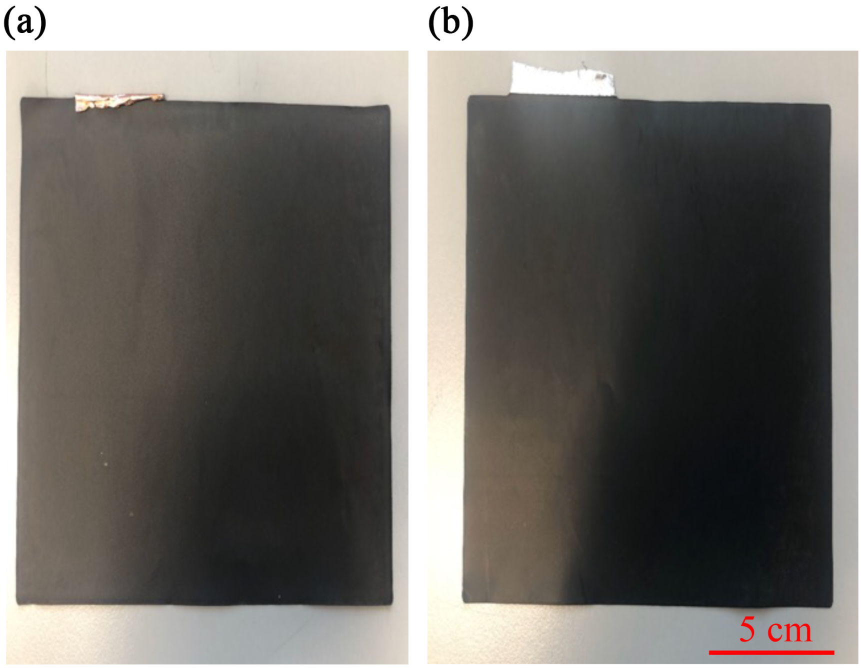

Figure S2. A single layer of electrodes extracted from the LFP pouch cell. (a) Cathode; (b) Anode.

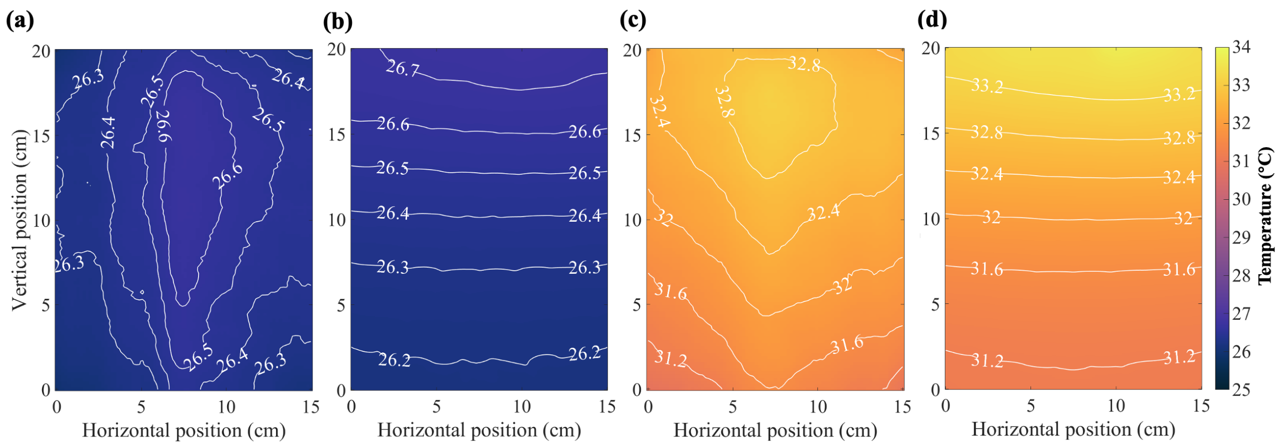

Figure S3. Thermal images of the LFP pouch cell at *t*=2500 s under different test conditions. (a) Square-wave cycling with 2C-100 s applied current at 30% SOC. (b) Simulation of (a) with the streamlined model.[1] (c) Square-wave cycling with 4C-100 s applied current at 30% SOC. (d) Simulation of (c) with the streamlined model.[1] The battery tabs (not shown) are on the top edge of the thermal images.

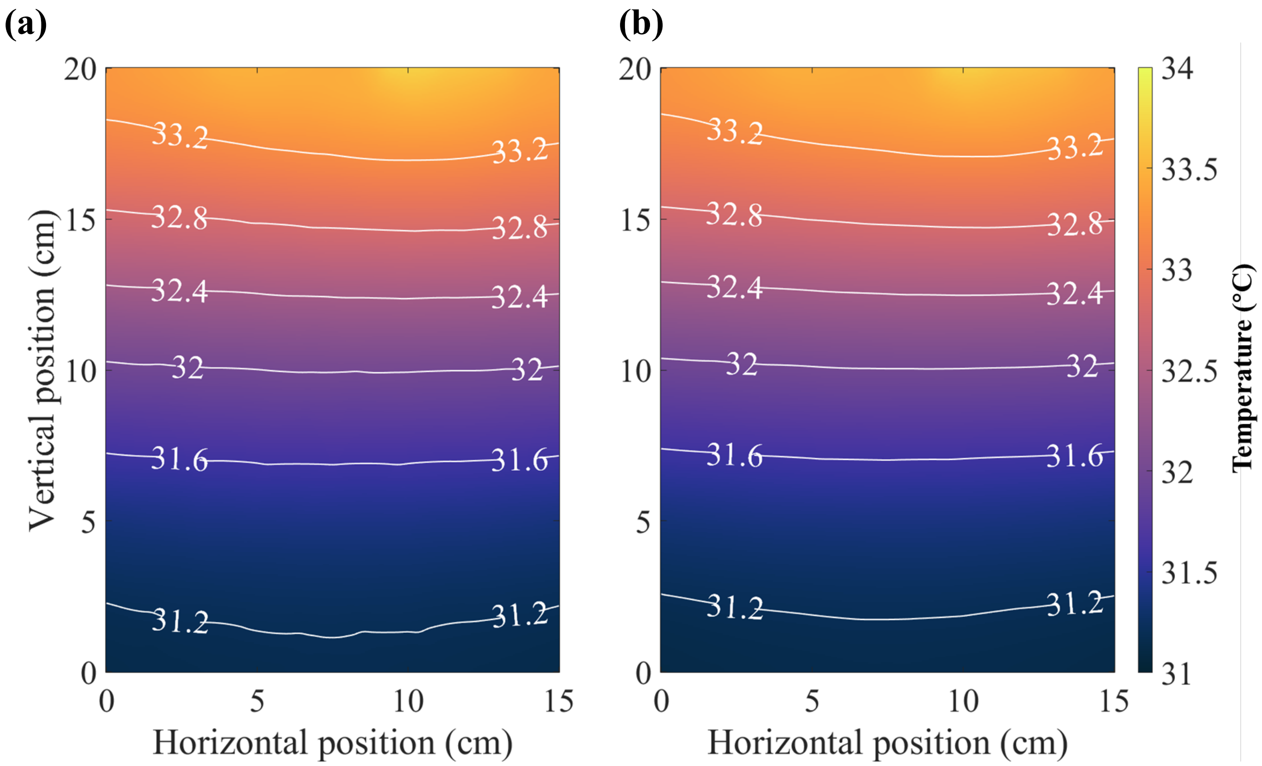

Figure S4. Temperature simulation results by the streamlined model[1] with different expressions describing reaction kinetics. (a) Linear kinetics; (b) Butler–Volmer kinetics. The simulated thermal images are at *t*=2500 s and 30% SOC with 4C-100 s applied current. The battery tabs (not shown) are on the top edge of the thermal images.

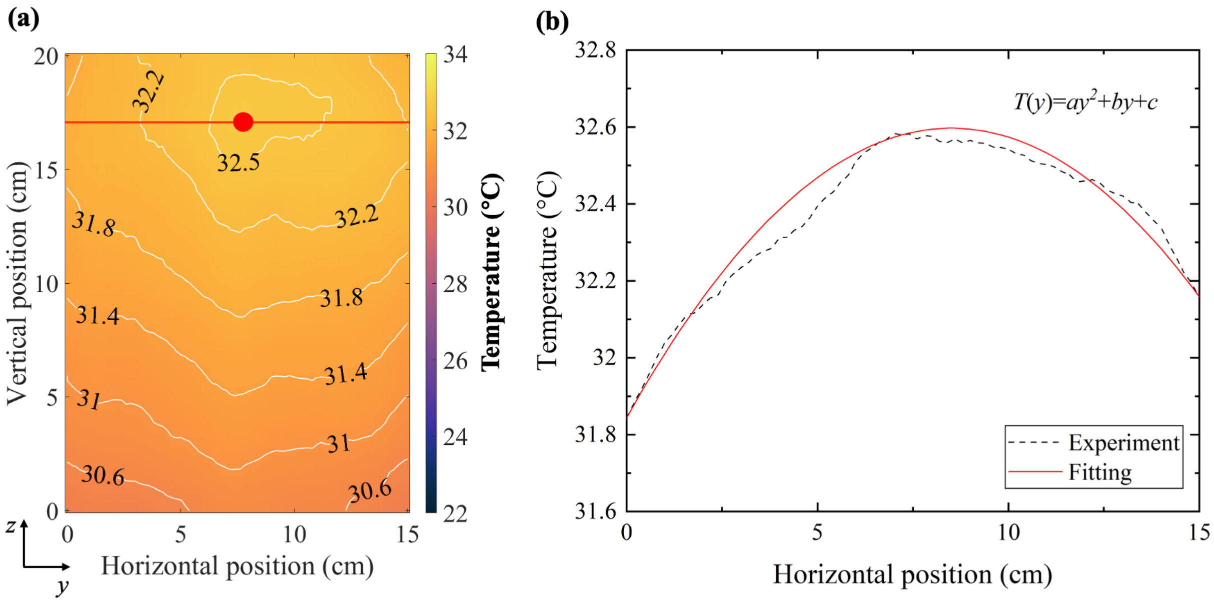

Figure S5. Computation of the cell horizontal temperature concavity from data. (a) Surface-temperature variation yielded by a thermography image at t=2500 s from a square-wave cycling test with 4C-50 s applied current at 30% SOC (tabs at the top of the image), showing the location of the hot spot (red •) and an axis passing through it parallel to the y axis (red –). (b) Fit of the temperature variation along the horizontal axis through the hot spot with a quadratic polynomial forced through the temperatures at the left and right edges and the hot spot. The horizontal temperature concavity relates simply to parameter a, as described below in Supplemental Note 1.

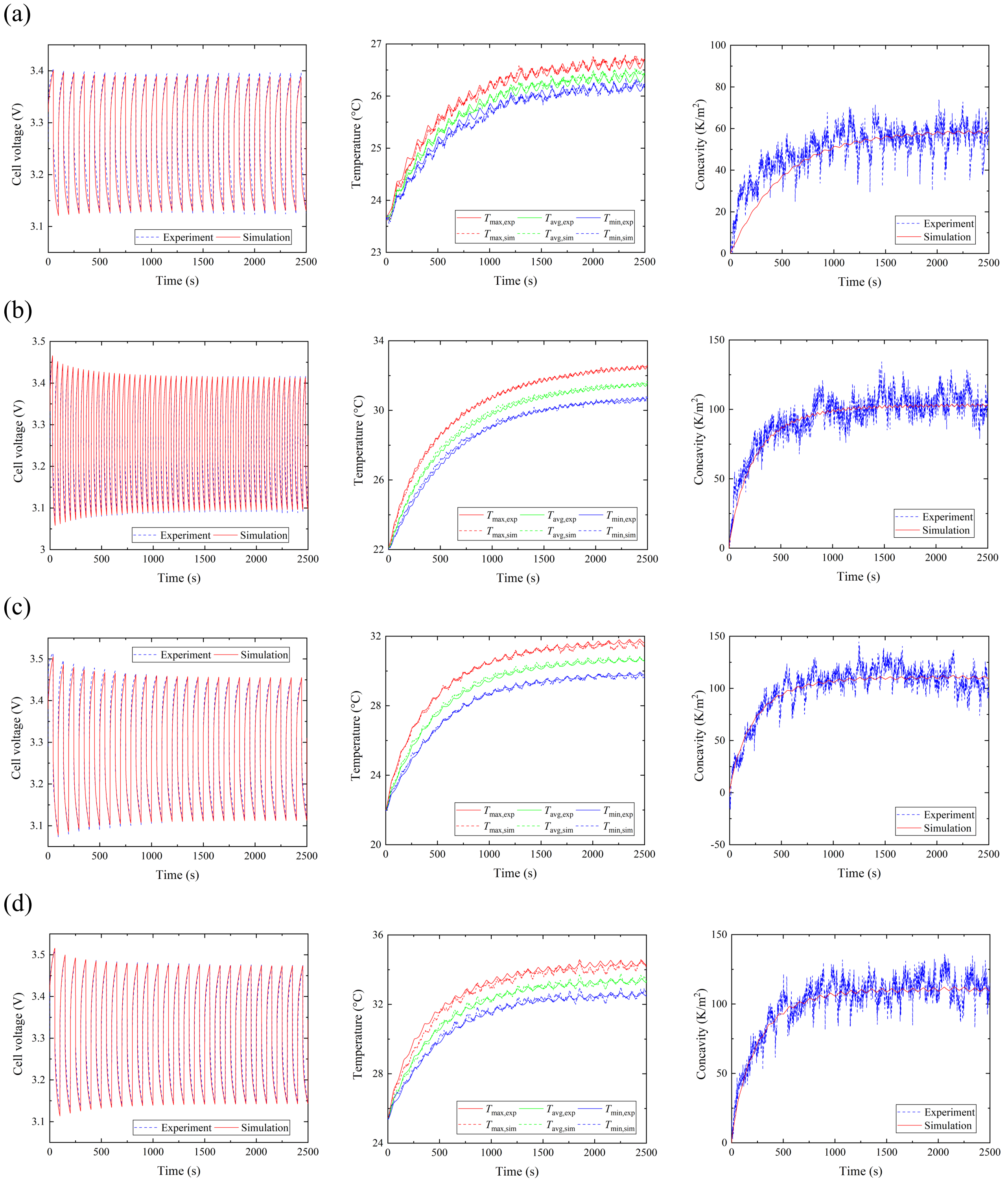

Figure S6. (a,b) Validation of parametrization yielded by the fits in Figure 3, showing cell voltage, surface temperatures, and horizontal temperature concavity measured and predicted under square-wave cycling at (a) 2C-100 s@30% SOC and (b) 4C-50 s@30% SOC cycling. (c,d) Data fits resulting from best fits of experimental data at (c) 4C-100 s@50% SOC and (d) 4C-100 s@70% SOC.

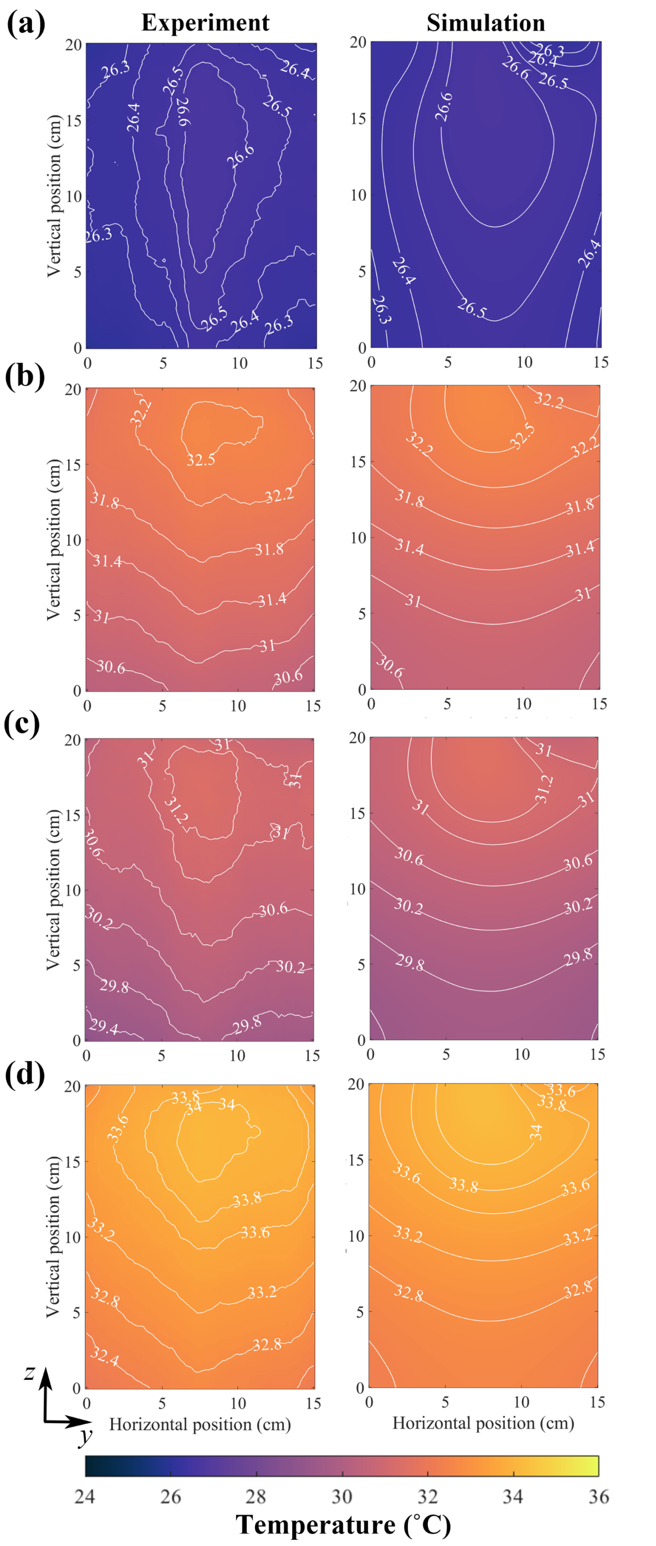

Figure S7. Experimental and simulated thermal images (*t*=2500 s) of LFP pouch cell with different applied current and SOCs. (a) 2C-100 s @30%SOC. (b) 4C-50 s @30%SOC. (c) 4C-100 s @50%SOC. (d) 4C-100 s @70%SOC. The battery tabs (not shown) are on the top edge of the thermal images.

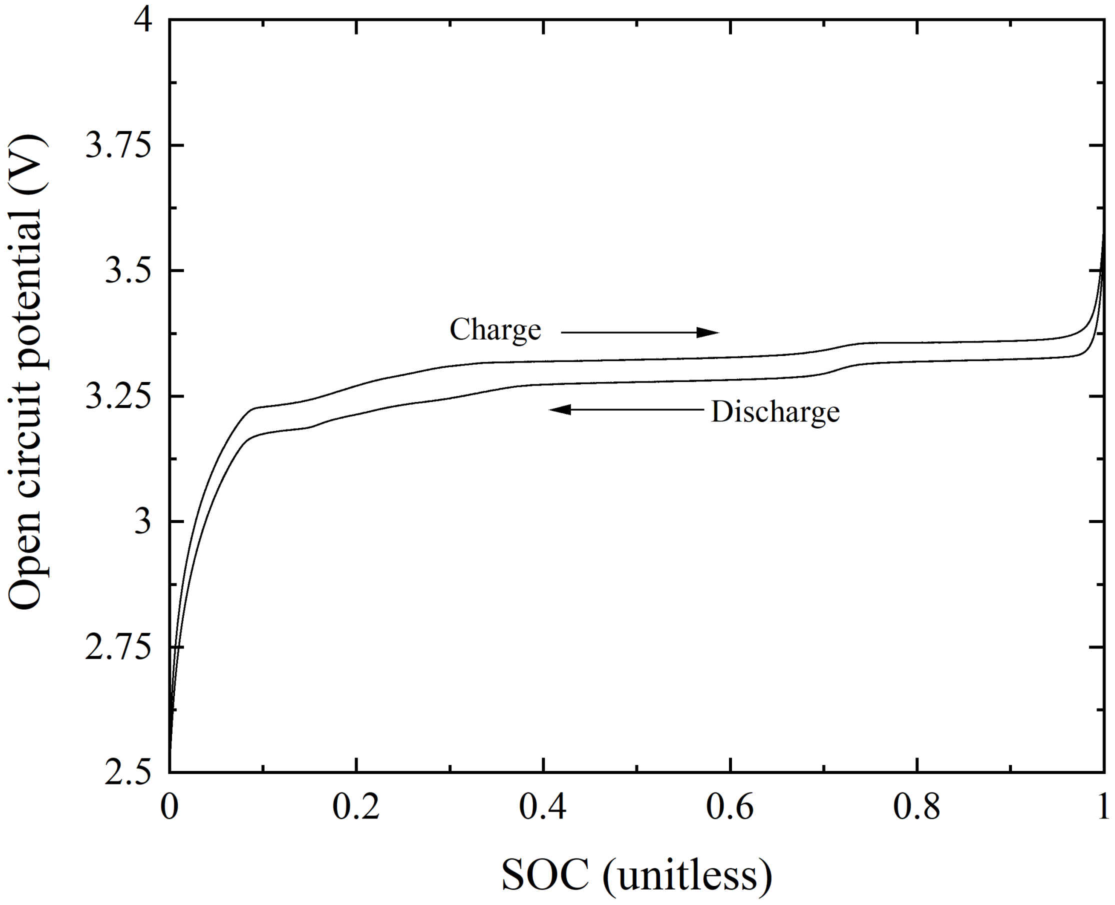

Figure S8. Pseudo-open circuit potential (OCP) of the LFP pouch cell, measured at C/25 constant current in a thermal chamber at 25.0 °C.

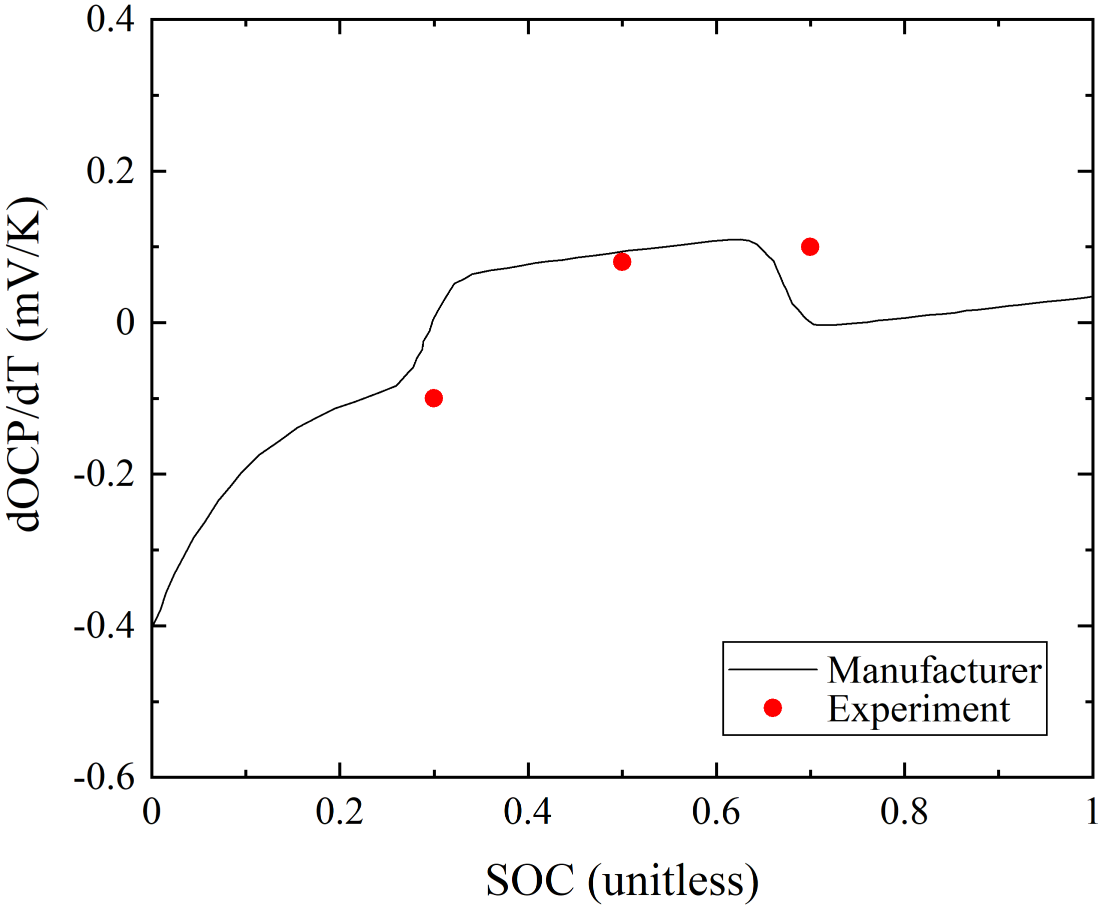

Figure S9. Temperature derivative of LFP pouch cell OCP. The experiment data points were obtained from square-wave cycling tests at 30%, 50% and 70% SOC.

Table S1 Specifications of the A123 LFP pouch cell.

Parameter	Symbol	Unit	Value
Battery length	L	mm	200
Battery width	W	mm	150
Battery thickness (excluding pouch thickness)	δ	mm	6.5
Tab length	L_{tab}	mm	25
Tab width	W_{tab}	mm	48
Tab thickness	δ_{tab}	mm	0.25
Positive current collector half-thickness	$\delta_{\mathrm{pe,cc}}$	μm	12.5
Positive electrode thickness	δ_{pe}	μm	70
Separator thickness	δ_{sep}	μm	20
Negative electrode thickness	δ_{ne}	μm	40
Negative current collector half-thickness	$\delta_{\mathrm{ne,cc}}$	μm	12.5
Number of stacked unit cells	N		42

Table S2 Fitted parameter sets gathered from four cells (from the same manufacturer lot) at 4C-100s@50% SOC. Cells are labelled I, D, E, and F; data in the main body are all from cell "I".

Parameter	Symbol	I	D	E	F	Avg.	Error (%)
Local reaction current density (A cm^{-3})	ai_0^θ	1.80	1.86	1.80	1.77	1.81	1.3
Reaction activation energy (kJ mol^{-1})	E^θ	29.2	28.8	29.0	29.1	29.0	0.3
Effective ionic conductivity (mS cm^{-1})	κ	0.431	0.553	0.428	0.426	0.460	6.8
Temperature coefficient of κ (mS m^{-1} K^{-1})	α	2.70	2.66	2.74	2.73	2.71	0.7
OCP gradient (V)	k_{U}	0.241	0.268	0.334	0.244	0.272	8.0
Diffusion time (s)	t_{d}	590	498	564	577	557	3.7
Entropy change (J mol^{-1} K^{-1})	ΔS	7.67	7.65	7.73	7.65	7.68	0.2
Heat transfer velocity (μm s^{-1})	$\frac{h}{C_p}$	5.06	4.90	4.90	4.95	4.95	0.8
Effective thermal conductivity (W m^{-1} K^{-1})	k	1.22	1.24	1.25	1.22	1.23	0.6

Table S3 Model parameters used for full-discharge simulations. Electrode porosity and particle radius were assumed identical for the positive and negative electrodes; values labelled 'assumed' are based on previous studies on similar cells[2] and are consistent with other reports from the literature.[3-5,7] The effective electrical and ionic conductivity, solid-state diffusivity, reaction current density, heat capacity and thermal conductivity obtained from the square-wave cycling tests were directly applied in the simulations.

Parameter	Symbol	Cathode	Anode	Method
Porosity (unitless)	ε_l	0.50	0.50	Assumed
Particle radius (μm)	r_0	5.0	5.0	Assumed
Charge capacity (Ah)	$\overline{Q}$	20.0		Manufacturer
Effective electrical conductivity (S/m)	σ	50		Fitted
Effective ionic conductivity (S/m)	κ	0.046		Fitted
Temperature coefficient of κ (mS/(m·K))	α	2.4		Fitted
Solid-state diffusivity (m^2/s)	D_s	4.5×10^{-14}		Fitted
Local reaction current density (A/m^2)	i_0^θ	6.0		Fitted
Reaction activation energy of i_0^θ (kJ/mol)	E^θ	29.5		Fitted
Heat capacity ($J/(m^3\cdot K)$)	C_p	2.35×10^6		Fitted
Effective thermal conductivity (W/(m·K))	k	1.1		Fitted
Heat transfer coefficient ($W/(m^2\cdot K)$)	h	12.0		Fitted

Supplemental Note 1. Horizontal temperature concavity

We call the 'horizontal direction', the direction perpendicular to the battery tabs and parallel to the separator (the direction of the y-axis in Figure S5 or S7). The effective horizontal temperature concavity was approximated by fitting the temperature distribution in the horizontal direction along an axis through the hot spot on the cell surface, that is, the temperature direction in the y-direction passing through the location of the surface-temperature maximum.

Effective concavity was estimated by assuming that the horizontal temperature profile through the hot spot could be approximated by a quadratic function,

$$T(y) = ay^2 + by + c. \tag{S1}$$

The coefficients in equation S1 were determined by forcing this function through three temperatures along the horizontal axis through the hot spot (cf. Figure S5a): the temperatures at the left and right edges, $T(0) = T_0$ and $T(L_y) = T_L$, respectively, and the temperature at the position of the hot spot, $T(y_{\text{hot}}) = T_{\text{hot}}$. The results of this fitting process show that the effective horizontal concavity of the temperature profile, k_c, is

$$k_c = \tfrac{1}{2}\left|T''(y_{\text{hot}})\right| = |a| = \frac{1}{y_{\text{hot}}\left(L_y - y_{\text{hot}}\right)}\left\{T_{\text{hot}} - \left[T_L \frac{y_{\text{hot}}}{L_y} + T_0\left(\frac{L_y - y_{\text{hot}}}{L_y}\right)\right]\right\}. \tag{S2}$$

Supplemental Note 2. Battery model

The original Doyle-Fuller-Newman (DFN) model considers charge and mass balances in both the solid and liquid phases within porous electrodes, with the heterogeneous reaction rate within the material calculated via Butler-Volmer kinetics, and the intercalation dynamics governed by spherical diffusion. The governing equations at interior points are written as follows:

Charge balance in solid: $$\nabla \cdot \vec{i}_s = -ai \tag{S3}$$

Charge balance in liquid: $$\nabla \cdot \vec{i}_1 = ai \tag{S4}$$

Mass balance in solid: $$\frac{\partial c_s}{\partial t} = \frac{D_s}{r^2}\frac{\partial}{\partial r}\left(r^2 \frac{\partial c_s}{\partial r}\right) \tag{S5}$$

Mass balance in liquid: $$\varepsilon \frac{\partial c_1}{\partial t} + \nabla \cdot \vec{N}_1 = \frac{ai}{F} \tag{S6}$$

Butler-Volmer kinetics: $$i = i_0\left[\exp\left(\frac{\beta F\eta}{RT}\right) - \exp\left(-\frac{(1-\beta)F\eta}{RT}\right)\right] \tag{S7}$$

where $\vec{i}_s = -\sigma\nabla\phi_s, \vec{i}_1 = -\kappa\nabla\phi_1 + \frac{\kappa_D RT}{F}\nabla \ln c_1, \vec{N}_1 = -D_1\nabla c_1 + \frac{\vec{i}_1 t_+^0}{F}$, and a Neumann boundary condition is used to relate the interfacial current density i to $\left(\partial c_s / \partial r\right)\big|_{t,r_0}$, along with a no-flux condition at the centre of the active particles. Here, $\vec{i}_s$ and $\vec{i}_1$ are the solid-phase and liquid-phase current density, a is the specific interfacial surface area within the porous electrode, and i is the reaction current per unit area of the pore surface; t is time, r is radial position within the intercalation particle at a given location, c_s is the molar concentration, and D_s is the solid-phase diffusion coefficient of lithium in the intercalation particle; F is Faraday's constant, ε is the electrode porosity, c_l is the liquid-phase salt molarity, and $\vec{N}_1$ is the superficial salt flux. In the kinetic rate law, i_0 is the exchange-current density, β is the symmetry factor, η is the surface overpotential, R is the universal gas constant and T is temperature. In the flux laws, ϕ_s and ϕ_1 are the solid-phase and liquid-phase electrical potentials, respectively; σ and κ are the effective electrical conductivity in the solid and liquid, respectively; κ_D is the effective conductivity associated with concentration overpotential (formally related to transference number and thermodynamic factor), D_1 is the liquid-phase Fickian salt diffusivity, and t_+^0 is the cation transference number relative to the solvent velocity. Last, r_0 is the average radius of the intercalation particles. Note that in simulations, the r direction is a pseudo-dimension orthogonal to the x, y, and z directions (cf. Figure S10 below) at every grid point, so that the model has four effective spatial dimensions.

When exchange-current density is large (or overpotential is small), the reaction kinetics can formally be linearized, yielding

$$i = i_0 \frac{F\eta}{RT}, \tag{S8}$$

a relation that is independent of the symmetry factor. If fast electrolyte diffusion is assumed in the liquid phase, then $D_1 \to \infty$, and liquid-phase concentration polarization can be neglected, such that

$$\nabla c_l = 0 \ . \tag{S9}$$

Accordingly, $\vec{i}_1 \approx -\kappa\nabla\phi_1$, equation (S9) becomes redundant, and the remaining balances and flux laws reduce to the Newman–Tobias porous-electrode model.[6] Rearranging the remaining balances in light of equations S8 and S9 gives the simplified battery model described in the Experimental Procedures.

It is also possible to obtain the prior streamlined model of Chu et al.[1] within this framework. If lithium diffusion in the solid particles is assumed to be fast, then

$$\frac{\partial c_s}{\partial r} = 0 \quad \text{and} \quad \frac{\partial c_s}{\partial t} = -\frac{ai}{F}, \tag{S10}$$

where the second result owes to the divergence theorem. Thus, the governing system becomes

Charge balance in solid: $$\nabla \cdot \vec{i}_s = -ai \tag{S11}$$

Charge balance in liquid: $$\nabla \cdot \vec{i}_l = ai \tag{S12}$$

Mass balance in solid: $$\frac{\partial \langle q \rangle}{\partial t} = -\frac{ai}{Fc_{\mathrm{s,max}}} \tag{S13}$$

Linear reaction kinetics: $$i = i_0 \frac{F\eta}{RT} \tag{S14}$$

In which equation S13 replaces equation S5. Here $\langle q \rangle$ is a new variable that expresses the average SOC of the intercalation particle and $c_{\mathrm{s,max}}$ is the maximum lithium concentration in the solid.

Supplemental Note 3. Model simplification via scaling analysis

Chu et al.[1] identify a set of dimensionless parameters whose values can be rescaled to replace a fully resolved multiple-layer model with a homogenized single-layer model. If the dimensionless quantities describing the fully resolved model should remain identical to those describing the homogenized model, then the following rescaling of parameters is required:

$$\sigma_1 = N^2\sigma_N, \quad \kappa_1 = N^2\kappa_N, \quad \sigma_{1,cc} = N^2\sigma_{N,cc}, \quad \kappa_{1,sep} = N^2\kappa_{N,sep}, \tag{S15}$$

in which the subscripts "1" and "*N*" represent the properties of the (homogenized) single-layer and (fully resolved) *N*-layer cell, respectively.

These rescalings were incorporated into the COMSOL Multiphysics implementation. Apart from the battery geometry, copper bars used as heat sinks connected to the tabs were also included in the model geometry, as shown below in Figure S10. The geometry was meshed and solved using COMSOL.

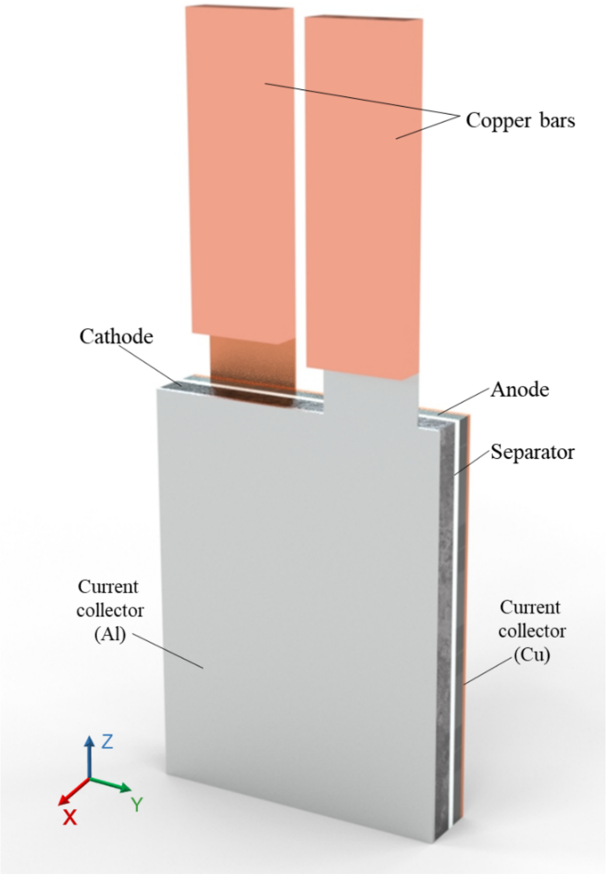

Figure S10. 3D single-layer battery geometry.

Supplemental Note 4. Model parameterization for full-discharge simulations

The pseudo-OCP of the LFP pouch cell was measured at 25 °C as a function of SOC by galvanostatic charge and discharge. Exemplary pseudo-OCP data are shown in Figure S8. In simulations, this OCP was assigned to the LFP cathode, while the graphite OCP was taken to be ground, i.e., the lithiated graphite at its average SOC was taken to be the thermodynamic reference for cell OCP.

Manufacturer data for the temperature derivative of OCP, shown in Figure S9, was used to obtain the SOC-dependent entropy change within the pouch cell, through

$$\Delta S(q) = F\left(\frac{\partial U}{\partial T}\right)_q . \tag{S16}$$

These data were validated using the entropy changes that resulted from fitting square-wave cycling tests at 30%, 50% and 70% SOC. After conversion to OCP derivatives using equation S16, these points were plotted on Figure S9 to illustrate the good agreement between the fitted entropies and the manufacturer data. In simulations, the entropy change due to the cell reaction was assumed to be divided equally between the positive and negative electrode.

Supplementary References

1. Chu, H.N., Kim, S.U., Rahimian, S.K., Siegel, J.B., and Monroe, C.W. (2020). Parameterization of prismatic lithium–iron–phosphate cells through a streamlined thermal/electrochemical model. *J. Power Sources* **453** 227787.
2. Gauthier, G. (2018). Experimental and modeling investigation of thermal behaviour and performance of lithium ion prismatic cells at cold-start temperatures (Ph.D. Thesis, University of Waterloo, Canada).
3. Gerver, R.E., and Meyers, J.P. (2011). Three-Dimensional Modeling of Electrochemical Performance and Heat Generation of Lithium-Ion Batteries in Tabbed Planar Configurations. *J. Electrochem. Soc.* **158** A835.
4. Kasavajjula, U.S., Wang, C., and Arce, P.E. (2008). Discharge Model for $LiFePO_4$ Accounting for the Solid Solution Range. *J. Electrochem. Soc.* **155** A866.
5. Kumaresan, K., Sikha, G., and White, R.E. (2008). Thermal Model for a Li-Ion Cell. *J. Electrochem. Soc.* **155** A164.
6. Newman, J.S., and Tobias, C.W. (1962). Theoretical analysis of current distribution in porous electrodes. *J. Electrochem. Soc.* **109**, 1183-1191.
7. Plett, G.L. (2015). *Battery Management Systems: Vol. 1. Battery Modeling* (Artech House, UK).
