# Supplementary material for: Multiscale coupling of surface temperature with solid diffusion in large lithium-ion pouch cells
Source: Commun Eng. 2022 May 26;1:1. doi: 10.1038/s44172-022-00005-8 (PMC10938860; doi:10.1038/s44172-022-00005-8)
Supplement: Supplementary file 5 — Description of Additional Supplementary Files [file 44172_2022_5_MOESM5_ESM.pdf]

Description of Additional Supplementary Files

File Name: Supplementary Movie 1

Description: Exemplary thermography data and simulation results during square-wave cycling. Transient surface-temperature distributions during a 4C—100s@30% square-wave cycling test.

File Name: Supplementary Movie 2

Description: Thermography data and simulation results during a full discharge at 4C.
